# Outer Electrospun Polycaprolactone Shell Induces Massive Foreign Body Reaction and Impairs Axonal Regeneration through 3D Multichannel Chitosan Nerve Guides

**DOI:** 10.1155/2014/835269

**Published:** 2014-04-09

**Authors:** Sven Duda, Lutz Dreyer, Peter Behrens, Soenke Wienecke, Tanmay Chakradeo, Birgit Glasmacher, Kirsten Haastert-Talini

**Affiliations:** ^1^Hannover Medical School, Institute for Neuroanatomy, Carl-Neuberg-Straße 1, 30625 Hannover, Germany; ^2^Institute for Multiphase Processes, Leibniz University Hannover, Callinstraße 36, 30167 Hannover, Germany; ^3^3Institute for Biophysics, Leibniz University Hannover, Herrenhäuser Straße 2, 30419 Hannover, Germany” for the sake of consistency Institute for Biophysics, Leibniz University Hannover, Herrenhäuser Straße 2, 30419 Hannover, Germany; ^4^Center for Systems Neuroscience (ZSN) Hannover, Hannover, Germany

## Abstract

We report on the performance of composite nerve grafts with an inner 3D multichannel porous chitosan core and an outer electrospun polycaprolactone shell. The inner chitosan core provided multiple guidance channels for regrowing axons. To analyze the * in vivo *properties of the bare chitosan cores, we separately implanted them into an epineural sheath. The effects of both graft types on structural and functional regeneration across a 10 mm rat sciatic nerve gap were compared to autologous nerve transplantation (ANT). The mechanical biomaterial properties and the immunological impact of the grafts were assessed with histological techniques before and after transplantation * in vivo*. Furthermore during a 13-week examination period functional tests and electrophysiological recordings were performed and supplemented by nerve morphometry. The sheathing of the chitosan core with a polycaprolactone shell induced massive foreign body reaction and impairment of nerve regeneration. Although the isolated novel chitosan core did allow regeneration of axons in a similar size distribution as the ANT, the ANT was superior in terms of functional regeneration. We conclude that an outer polycaprolactone shell should not be used for the purpose of bioartificial nerve grafting, while 3D multichannel porous chitosan cores could be candidate scaffolds for structured nerve grafts.

## 1. Introduction

Trauma patients are often affected by injuries to the peripheral nervous system [1]. The associated sensory and motor defects, as well as neuropathic pain syndromes, can lead to devastating life-long functional disability and socioeconomic burden [2]. In case of complete transection of a peripheral nerve with severe loss of nervous tissue between the dehiscent nerve ends, microsurgical nerve autotransplantation remains the standard therapy [3, 4]. However, as complete functional recovery is seldom achieved and autologous nerve transplantation is accompanied by donor-site morbidity and limited availability, treatment using bioengineered nerve guidance channels (NGCs) is a promising therapeutic alternative [3–5]. A large number of studies investigating different biological, synthetic nonbiodegradable and biodegradable biomaterials with custom-tailored graft properties have been performed in order to develop a nerve graft that best mimics the environment of a regenerating peripheral nerve [5]. Scaffolds used for nerve reconstruction have to be biocompatible and biodegradable to allow optimal integration into the nervous tissue. Their mechanical solidity should be tailored to match the regeneration progress [6], which is only possible as soon as the regeneration rates have been evaluated for a specific nerve and graft type. Additionally the artificial NGCs have to permit nutrient exchange while prohibiting extensive infiltration of scar tissue [6]. Two cell types have to be supported during peripheral nerve regeneration: neurons and peripheral glia cells (Schwann cells—SCs). Molecular alterations and the interaction between these cells play a key role in axonal outgrowth and guidance into the target tissue [5, 7].

The property of longitudinally aligned microchannels within NGCs has proven to be beneficial for peripheral nerve regeneration and can be achieved by directional solidification of polymer solutions [8]. While others worked with collagen based scaffolds, we focus on chitosan, a copolymer derived from deacetylation of chitin. It is composed of d-glucosamine and N-acetyl-d-glucosamine and thus allows interactions with the extracellular matrix in nervous tissue [6]. Chitosan has an outstanding SC affinity facilitating SC migration, adhesion, and longitudinal arrangement and therefore it is a promising biomaterial for peripheral nerve repair [9]. The excellent suitability of NGCs made of chitosan has already been proven [10–12].

Polycaprolactone (PCL) belongs to the class of aliphatic polyesters and is a synthetic polymer synthesized by ring opening polymerization [13]. PCL has shown favorable influence on glia cell proliferation, migration, adhesion, axonal orientation, and outgrowth [14]. Due to the possibility of tailoring the degradation rate and mechanical properties of aliphatic polyesters, PCL has been reported to be highly qualified for utilization in artificial nerve grafts [6]. Polycaprolactone NGCs have also already been investigated as a potential alternative to autologous nerve grafts [15, 16].

In the present study we investigated the applicability of composite NGCs made of a 3-dimensional, multichannel, porous chitosan core sheathed with an electrospun PCL coat (COM) in the model of 10 mm sciatic nerve defect reconstruction in adult rats. Although this gap length is not a critical defect for functional recovery, it is a standard model to assess NGCs properties in general [12]. As the isolated chitosan cores could not be sutured directly to the nerve ends, they were implanted within the epineural sheath of sciatic nerves (10 mm gap) as control group (CES). To evaluate the biocompatibility of the COM and CES grafts as well as their properties with regard to peripheral nerve reconstruction and regeneration, histological, functional, and nerve morphometric data were obtained and compared to data obtained after autologous nerve transplantation (ANT) in another control group.

## 2. Materials and Methods

### 2.1. Chitosan Core Manufacturing

**Figure 1:**
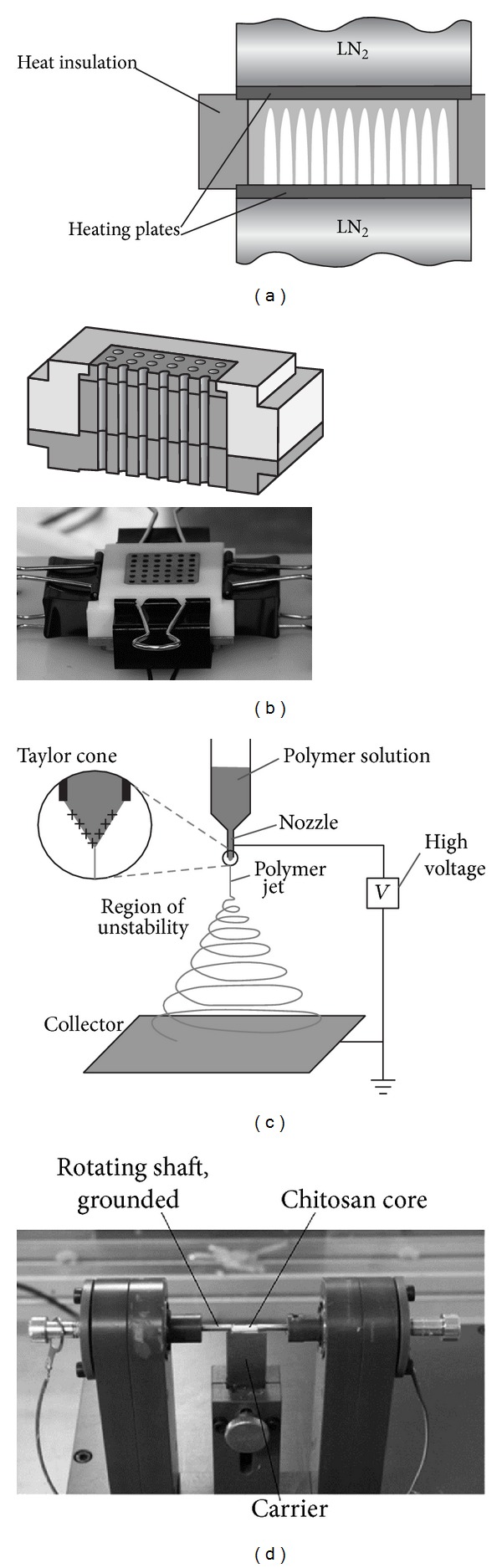
Fabrication of the CES and COM grafts. (a) In the power-down technique, unidirectional crystal growth in a chitosan solution is achieved by freezing at a defined cooling rate with a maintained constant local temperature gradient. The temperature is controlled by two heating plates, which are in contact with liquid nitrogen (LN_2_). The crystals exclude chitosan and, after freeze drying, leave behind longitudinal pores with intact chitosan walls. (b) Experimental chitosan cores were produced in a three-part copper mold with Teflon insulation. The mold produces 36 chitosan cores at the same time. For open, longitudinal pores, upper and lower parts were sheared off after freezing. (c) Principle of electrospinning: a polymer solution is slowly pumped through a nozzle and gets charged into the high voltage electrical field. Due to the positive charge accumulated in the polymer drop, a Taylor cone forms with an emerging polymer jet at the tip. With continuous evaporation of the solvent, the jet solidifies into a polymer fiber that is randomly deposited on a grounded collector. This collector can have any shape, determining the shape of the product. (d) Rotator for assembly of the composite graft (COM) sheath. By grounding the shaft, electrospun fibers are deposited on chitosan cores. A PCL polymer solution was spun at 24–28 kV with a flow rate of 0.5 mL/h at a distance of 21 cm to the collector. After initial spinning for 30 s on two opposite sides, the carrier was removed and the chitosan core was spun in a rotational manner for 4 min.

The 3-dimensional, multichannel chitosan cores were produced using the “power-down” directional solidification technique [17–19]. The crystallization of chitosan solution in directional solidification occurs in a defined manner in order to control unidirectional crystal growth. The medium is subjected to a constant temperature gradient (*G*) across the chitosan solution (Figure 1(a)) while cooling down at a defined cooling rate (*B*). This is achieved by placing the solution between two compartments that are cooled using liquid nitrogen. The temperatures of the surfaces that are in contact with the solution are separately adjustable by heated plates.

The crystal growth velocity is a function of cooling rate and temperature gradient (*v* = *B*/*G*). During freezing, chitosan is excluded from the growing dendritic ice crystals. It concentrates and gets compressed, thus forming longitudinal channels of ice walled by chitosan. Removal of ice by freeze-drying leaves behind a porous structure while still retaining the prior structure and arrangement of chitosan [20, 21].

A higher crystal growth velocity *v* results in crystals of smaller diameters and thus smaller pore diameters in the final product [17–19]. This basic phenomenon has been used to produce nerve guides with unobstructed, unidirectional, end-to-end pores.

For production of the 3D-multichannel chitosan cores, 2.5% (w/v) medium molecular weight chitosan (190,000–310,000 g/mol, Sigma Aldrich, Deisenhofen, Germany) with a deacetylation degree of 75–85% was dissolved in 1% acetic acid. The resulting highly viscous and transparent solution was transferred into the 36 cylindrical cavities of an insulated, tripartite mold (Figure 1(b)). The cavities of the center pieces were 10 mm long with a diameter of 2 mm. The filled molds were transferred into the power-down apparatus to be directionally solidified at a cooling rate of *B* = 3.0 K/min and a constant spatial temperature gradient of *G* = 1.5 K/mm across the mold. After solidification the upper and lower parts of the mold were sheared off. The molds containing the solidified solution were directly freeze-dried with primary and secondary drying at 0.35 mbar and 0.001 mbar, respectively. Finally samples were irradiated with UV light for 1 hour.

### 2.2. Polycaprolactone Shell Manufacturing

Electrospinning is the fabrication of thin fibers from a polymer solution by a high voltage electric field [22, 23]. Briefly, at very low flow rates, a polymer solution is pumped through a small nozzle. Upon exposure to an electric field, the charged polymer solution is drawn towards the grounded collector forming a thin continuously elongating fiber jet. The solvent dries during the flight phase and produces a continuous fiber mat on the collector (Figure 1(c)).

**Figure 2:**
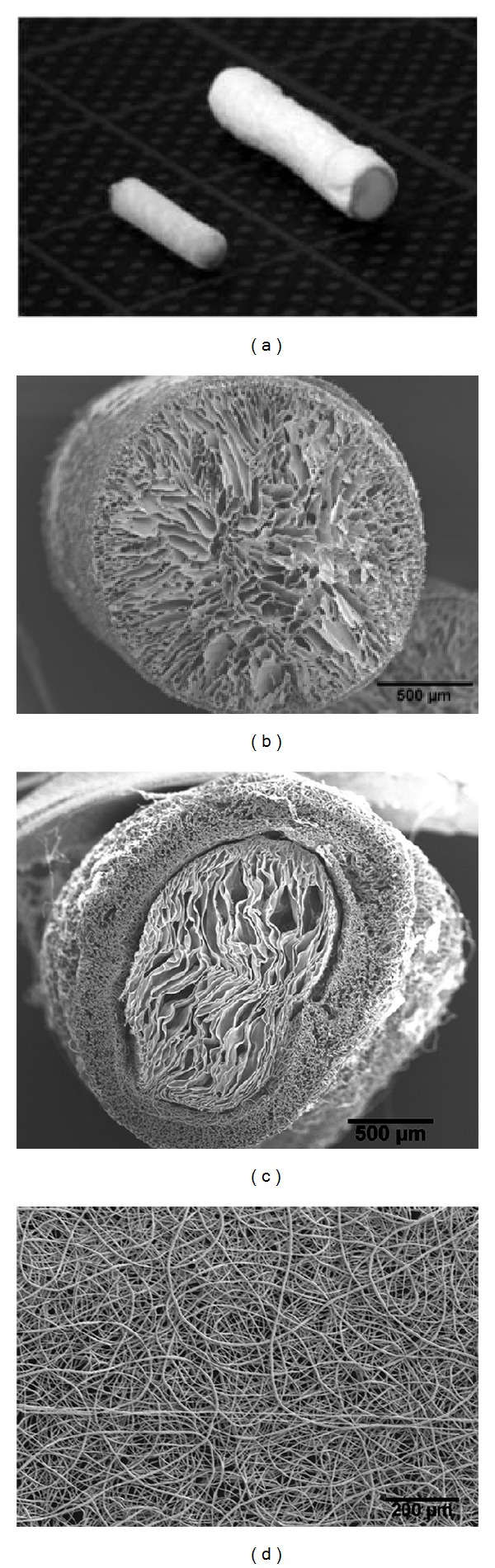
Photomicrographs of CES and COM grafts. (a) On the left an isolated chitosan core (CES graft) and on the right a COM graft are shown (grid length 10 mm). ((b)–(d)) Electron micrographs of the pore structure in cross-sections of the isolated chitosan core (b) or the COM grafts (c) and of the porous surface of the PCL-nanofiber shell of a COM graft ((d), top view).

For the manufacturing of the COM graft the prefabricated chitosan cores were placed in a specially designed collector, where only the axis is electrically conductive and grounded (Figure 1(d)). In the present study, a polymer solution of 20% PCL (70,000–90,000 g/mol, Sigma Aldrich, Deisenhofen, Germany) in chloroform/hexane (3 : 1) was spun at 24–28 kV at a flow rate of 0.5 mL/h and a distance of 21 cm. The two opposite sides of the chitosan cores were successively electrospun for 30 seconds to attach the core to both shafts. After this, the carrier was retracted and the cores were electrospun for 4 min on constant rotation to obtain a uniform deposit of the fibers forming the outer shell. Afterwards, the PCL fiber shell was trimmed leaving an extension of 2 mm on each side. Subsequently, the samples were irradiated with UV light for 1 hour.  Figure 2(a) shows representative photographs of a CES and a COM graft.

Additionally, tensile tests were carried out to evaluate the stability of the surgical connection. COM grafts were sutured to sciatic nerve ends in situ and immediately removed, leaving about 1 cm of the nerve ends. After storage in Hanks' solution for 24 h, the tensile tests were carried out with nerve ends directly clamped into the tensile testing machine (Series 5565 A, Instron, Pfungstadt, Germany). The tension stability was tested at a traverse speed of 10 mm/min.

### 2.3. Evaluation of Graft Properties by Scanning Electron Microscopy

The surface structure and the pore morphology of the CES and COM scaffolds were examined using a scanning electron microscope (VP-SEM S-3400 Type II, Hitachi High-Technologies Europe GmbH, Krefeld, Germany). Sample chitosan scaffolds produced at varying cooling rates (*B* = 1–5 K/min) and temperature gradients (*G* = 1, 1.5, 2.0 K/mm) were analyzed with respect to their pore morphology. The evaluation was performed using a user plugin (McMaster Biophotonics Facility (MBF) ImageJ for Microscopy, McMaster Health Science Center University, Ontario, Canada) for the open source image processing program ImageJ [24].

### 2.4. Experimental Design and Animal Surgery

In order to analyze the performance of the composite nerve grafts (COM group) as well as the bare chitosan cores implanted into the epineural sheaths (CES group), short- (2 weeks) and long-term observation (13 weeks) periods were chosen to compare the artificial grafts to autologous nerve transplants (ANT group). The experimental design and the number of animals evaluated are summarized in Table 1. Histological analysis and material assessment were performed in two animals per group at two and thirteen weeks after surgery. Functional evaluation was carried out continuously during the 13 weeks' observation period, after which nerve morphometry was subsequently evaluated.

Thirty-six adult female Wistar rats (approximately 220 g, 8 weeks, Janvier, Le Genest St. Isle, France) were kept as a foursome in Makrolon Type IV cages under standard conditions (temperature 22 ± 2°C, humidity 55 ±  5%, light/dark cycle 14 : 10 h) with food and water* ad libitum*. The animals were divided in three experimental groups with *n* = 4 animals in each group subjected to histological evaluation and *n* = 8 animals in each group subjected to evaluation of nerve recovery. For surgery, animals were anaesthetized by intraperitoneal injection of chloral hydrate (370 mg/kg body weight, Sigma Aldrich, Deisenhofen, Germany). Sufficient analgesia was ensured by intramuscular application of buprenorphine (0.045 mg/kg body weight, Buprenovet, WDT, Germany). The animals' left hind limb was shaved and the skin was disinfected. Animals were placed onto an electric heating pad and body temperature was monitored regularly. Using aseptic techniques, the left sciatic nerve was exposed by a skin incision along the femur followed by intermuscular approach. The nerve was dissected microsurgically from surrounding connective tissue and transected at a defined point at mid-thigh (2 mm distal of the muscle aponeurosis which is crossed by the nerve at the proximal thigh).

For autologous nerve transplantation (ANT), the sciatic nerve was transected for a second time 10 mm distal to the proximal transection site. The segment was reversed and sutured back into place using four epineural single interrupted sutures (9-0 Ethilon, Johnson & Johnson Medical GmbH, Norderstedt, Germany) at each coaptation site.

The composite graft implantation (COM) between the proximal and distal nerve ends was performed after resecting a 5 mm segment of the distal sciatic nerve. One single interrupted 9-0 Ethilon suture pulled the nerve 2 mm into the proximal and distal end of the transplant and fixed it, leaving a 10 mm gap.

For chitosan core implantation (CES), the epifascicular epi-/perineurium was cut in a longitudinal direction and the fascicles were resected over a distance of 10 mm leaving an empty epineural sleeve [25, 26]. The chitosan core was then implanted and the epifascicular epi-/perineurium was closed using single interrupted 9-0 Ethilon sutures.

Finally muscle layers and skin were closed with sutures. The animals were frequently checked for automutilation behaviour and antibite spray or rat collars were applied when necessary. Animal care, housing, surgery, and postoperative treatment were conducted in accordance with the German law on the protection of animals and were approved by the local animal care committee (LAVES 33.12_425502-04-12/0816).

### 2.5. Histological Evaluation of Immunological Events

Graft sites that were explanted for evaluation of the inflammatory response by hematoxylin/eosin staining and immunohistochemistry were resected inside the graft 2 mm proximal to the distal coaptation site of the reconstructed nerve (2 and 13 weeks after nerve reconstruction, *n* = 2 each group and point of time). The nerve ends were fixed in 4% PFA and stored at 4°C over night. Afterwards, specimens were dehydrated in an ascending ethanol series. The tissue was then treated with isopropanol for at least 1 h at room temperature followed by 30 minutes at 60°C and transferred into a 1 : 1 mixture of isopropanol and paraffin overnight at 60°C. Finally the tissue was immersed in paraffin at 60°C for 24 h and then paraffin blocks were molded before 7 *μ*m slices were cut. Hematoxylin/eosin (HE) staining was performed on four slices per animal, which were then observed under a computer-assisted BX51 microscope with cellSense Dimension software (Olympus, Hamburg, Germany). Digital images were made under varying magnifications for documentation of the presence of multinucleated foreign body giant cells (FBGCs).

Consecutive slices were processed for immunohistochemistry by twofold immersion in xylene for 10 min and then placed in distilled water via a descending ethanol series.

Finally, anti-ED1 (anti-CD68, 1 : 1000 in PBS plus 5% rabbit serum; Serotec, Düsseldorf, Germany) and anti-neurofilament 200 double staining (1 : 200 in tris-buffered saline; Sigma, Taufkirchen, Germany) were performed. As secondary antibodies Alexa-555 goat-anti-mouse and Alexa-488 goat-anti-rabbit were used (1 : 1000; Alexa, Invitrogen, Life Technologies GmbH, Darmstadt, Germany). DAPI (1 : 2000 in PBS; Sigma Aldrich, Deisenhofen, Germany) staining was added to mark all nuclei. Fourteen digital images were acquired from two ED1/NF200 stained slices per animal at +7 *μ*m and +147 *μ*m distal to the resection point with a computer-assisted IX-70 microscope and cell^∧^P software (Olympus, Hamburg, Germany). It has been shown that ED1 is a cytoplasmic antigen expressed by a phagocytotic phenotype of macrophages and monocytes [27, 28]. Four pictures represented the peripheral region of the graft and three pictures were made in the graft center to best analyze inflammatory cell infiltration in both slices, respectively. Cell counting was performed using ImageJ software and the mean number of ED1+ cells per mm^2^ was calculated from the given data.

### 2.6. Imaging of Explanted Biomaterial

Transplant center pieces (central 6 mm of of the grafts, 2 and 13 weeks after nerve reconstruction, *n* = 2 each group and point of time) were fixed in 4% formaldehyde for 24 h and cryo-sectioned into 50 *μ*m slices using a Mikrom HM 550 (Thermo Scientific, Waldham, USA). Samples were either postfixed in 1% OsO_4_, freeze-dried and sputtered with gold-palladium for scanning electron microscopy (S-3400N, Hitachi, Tokyo, Japan), or were stained for actin and nuclei (Phalloidin-Atto 488, Hoechst, Sigma Aldrich, Deisenhofen, Germany) and visualized using fluorescence microscopy (Axiovert 200, Carl Zeiss Imaging Solutions GmbH, Jena, Germany).

### 2.7. Pain Perception Testing (Pinch-Test)

Starting with the 4th week after surgery mechanoceptive pain perception recovery was weekly examined by pinching the most distal portion of toe 2 to 5 on the operated hind limb with an anatomic forceps. Pinching the distal phalanx with a flattened forceps results in withdrawal or vocalization in case of functional hind paw innervation [29]. Examinations were performed by the same, blinded investigator during all trials to ensure constant pressure and comparable pinching technique [30]. Due to saphenous nerve innervation of the two most medial digits, toe 2 served as positive control [29]. Due to anatomical proximity, reinnervation by regrowing sciatic nerve fibers was expected to occur first in toe 3 and last in toe 5.

### 2.8. Evaluation of Motor Function: Static Sciatic Index

The recovery of motor function was quantified weekly from week 4 to 13 by the static sciatic index (SSI). The SSI evaluates muscle reinnervation and function via changes in interdigital distances of the operated and nonoperated hind limb [31]. The animals were placed into a transparent Plexiglas box (20 × 12 × 9 cm) to acquire images of their plantar surfaces by a computer-assisted camera device [32, 33]. The toe spread (TS, distance between toe I and toe V) and intermediate toe spread (ITS, distance from toe II to IV) were measured for the operated (OTS/OITS) and nonoperated extremity (NTS/NITS) by digital image analysis (AxioVision, Carl Zeiss Imaging Solutions GmbH, Jena, Germany) to calculate the SSI according to the following formula [32, 33]:

(1) SSI=108.44∗(OTS−NTSNTS)+31.85∗(OITS−NITSNITS)−5.49.

### 2.9. Electrodiagnostic Measurements

The regeneration progress was evaluated by electrodiagnostic measurements at 13 weeks after surgery. Therefore compound muscle action potentials (CMAPs) of the gastrocnemius muscle were recorded using a computer-assisted portable electrodiagnostic device (Keypoint Portable, Medtronic Functional Diagnostics A/S, Skovlunde, Denmark). Anesthesia and monitoring were implemented as described above (see Section 2.4.). Due to death of one animal of the ANT and CES group each and only 6 measurable CMAPs in the COM group animal numbers for these recordings were as follows: ANT and CES *n* = 7; COM *n* = 6; and contralateral healthy nerve, *n* = 20. After microsurgical dissection of the nerves a bipolar steel hook electrode was used to stimulate the nerve twice, proximal and distal to the graft. The CMAPs were recorded in tendon-belly technique and examinations were performed as described elsewhere [32]. Exact placement of the recording as well as the stimulation electrodes is of high importance because costimulation of the proximal muscle branch of the sciatic nerve or corecording from neighboring muscles could result in false positive signals mostly from the biceps femoris muscle [34]. In order to avoid costimulation of the proximal muscle branch of the sciatic nerve we dissected it prior to proximal stimulation whenever it was located in close proximity to the nerve graft. For analysis of the electrophysiological properties, the nerves were stimulated with a current intensity of 1 mA [32]. The electrodiagnostic device automatically calculated the motor nerve conduction velocity (NCV) between the proximal and distal stimulation site from latency difference and distance between the stimulation points (mean distance: 18 mm).The percentage axonal loss (AxL) of the grafted nerve was calculated from the area under the curve (AUC) of the negative peak of the CMAPs recorded from both, the operated (*O*) and the normal (*N*) nerve, according to the following formula [32, 33, 35]:

(2) $$\begin{array}{c} \text{AxL}=\frac{N-O}{N}*100. \end{array}$$

The measurements provided information on the number of functional axons, their diameter, and degree of myelination for detecting differences in the regenerative status between the three reconstruction approaches [32, 36]. To further determine latency and current threshold for the first measurable CMAP as well as CMAP amplitude, latency, and current intensity for the evocable maximal CMAP (M CMAP amplitude), the nerves were stimulated with continuously increasing current intensity from 0.1 mA until the CMAP shape and amplitude did not change any longer [32]. A current intensity of 8 mA was not exceeded to avoid erroneously short CMAP latencies and muscle contraction by current spread [32, 36].

### 2.10. Harvest and Processing of Nerve Tissue for Morphometry

The graft site was explanted before the animals were sacrificed for muscle tissue harvest. Distal nerve segments harvested for morphometric analysis at +10.5 mm to the proximal coaptation site were fixed in a fixative according to Karnovsky (2% paraformaldehyde, 2.5% glutaraldehyde in 0.2 M sodium cacodylate buffer, pH 7.3) for 24 hours and rinsed three times with 0.1 M potassium dichromate containing 7.5% sucrose before postfixation in 1% OsO_4_ was performed for 1.5 hours [30, 32, 37]. Myelin staining was done by a modified protocol in potassium dichromate (1%, 24 h), ethanol (25%, 24 h), hematoxylin (0.5% in 25% ethanol, 24 h), and ethanol (25%, 24 h), as described previously [32]. After dehydration, nerve segments were embedded in epon resin and semithin sections (1 *μ*m) were cut. For morphometry, myelin staining was augmented with toluidine blue. The segment of the reconstructed nerve chosen for morphometric analysis was located distal to the grafts in order to analyze only axons that have successfully passed the graft at 13 weeks after surgery.

Finally, images of the entire nerve cross-sections of the distal nerve (+0.5 mm to the distal coaptation side) were acquired by automatic multiple-image-alignment process with the use of a computer-assisted Olympus BX51 microscope and cellSense Dimension software (Olympus, Hamburg, Germany). The images were analyzed using AnalySIS Pro (Olympus, Hamburg, Germany) [30, 32, 37]. Nerve cross-sections were analyzed stereologically and measurements were in accordance with the rules for coping with sampling errors in histomorphometry [38]. The two-dimensional dissector method (subarea 150 × 150 *μ*m, subfields 50 × 50 *μ*m) was used to determine the number of myelinated axons per cross-sectional area, nerve cross-sectional area, nerve fiber density, nerve fiber diameter, and nerve fiber area [39]. The *g*-ratio and myelin thickness were estimated from 4 grid pattern measurements, each with an area of 100 × 100 *μ*m, representing ≥200 axons [37]. The observer was blinded for all images to avoid information bias. Nerve fiber density was calculated by the following formula:

(3) $$\begin{array}{c} \text{Nerve}\;\;\text{fiber}\;\;\text{density}=\frac{\text{Number}\;\;\text{of}\;\;\text{axons}}{\text{Nerve}\;\;\text{cross-sectional}\;\;\text{area}}. \end{array}$$

### 2.11. Muscle Weight Ratio

Muscle weight was investigated to provide information on reinnervation by muscle tissue atrophy quantification [40]. Both, the gastrocnemius and anterior tibial muscle, were resected from the experimental (mw_*e*_, g) and untreated (mw_*u*_, g) lower leg and the explanted muscles were weighed immediately to calculate the muscle weight ratio (MWR) according to the formula:

(4) $$\begin{array}{c} \text{MWR}=\frac{{\text{mw}}_{e}}{{\text{mw}}_{u}}. \end{array}$$

### 2.12. Statistical Analysis

All data are given as mean ± SEM. Results were analyzed for significant differences by parametric or nonparametric one-way analysis of variance unless otherwise denoted in the results section. A *P* value of <0.05 was considered to indicate statistically significant differences between groups. For statistical analysis and graphical illustration GraphPad InStat (V 3.06) and GraphPad Prism (V 4.03) were used.

## 3. Results

### 3.1. Evaluation of the Initial Graft Properties by Scanning Electron Microscopy

The analysis of the SEM images of the chitosan cores showed that the average pore size and mean minimum pore size only slightly differ when the cooling rate is varied from *B* = 1–5 K/min and the temperature gradient from *G* = 1, 1.5, 2.0 K/mm. The average pore size area (cross-sectional area) of these samples could be estimated with reasonable accuracy up to 2,100 *μ*m², which corresponds to an equivalent circle diameter of 51 *μ*m. The average minimum pore diameter was within the range of 36–38 *μ*m. The shape of the pores was thus more oval than circular. Cross-sections through the final chitosan cores showed pores with an elongated, almost oval form (Figures 2(b) and 2(c)). The pore sizes were determined to an equivalent circle diameter of about 30 to 110 *μ*m.

The thickness of the PCL shell depended on the spinning time. At a spinning time of 2 min an average wall thickness of 174 *μ*m, at 5 min of 214 *μ*m and after 10 min of 250 *μ*m, was measured. The fiber diameter decreased from 8.1 ± 2.0 *μ*m to 5.7 ± 1.2 *μ*m by increasing the distance between nozzle and collector from 20 cm to 30 cm and the voltage from 21.6 kV to 32.4 kV. The PCL sheath used in the* in vivo* tests had a thickness of 214 ± 26 *μ*m and the diameters of the PCL fibers deposited on the chitosan scaffolds averaged 7.4 ± 0.8 *μ*m. The results of the SEM analysis showed that for nonrotating spinning the PCL fibers were deposited so densely that an average mesh size of 22 *μ*m² (60 s) and 9 *μ*m² (90 s) with a minimal medium mesh diameter of 2.4 *μ*m (60 s) and 1.8 *μ*m (90 s) occurred (see SEM image, Figure 2(d)). Tensile testing of the COM graft revealed that the suture between nerve end and PCL shell was the weak spot. At a tensile force of 0.25 ± 0.03 N, the suture was ruptured at the epineurium while the PCL shell was still intact.

### 3.2. Characterization of Inflammatory Reactions to the Grafts

**Figure 3:**
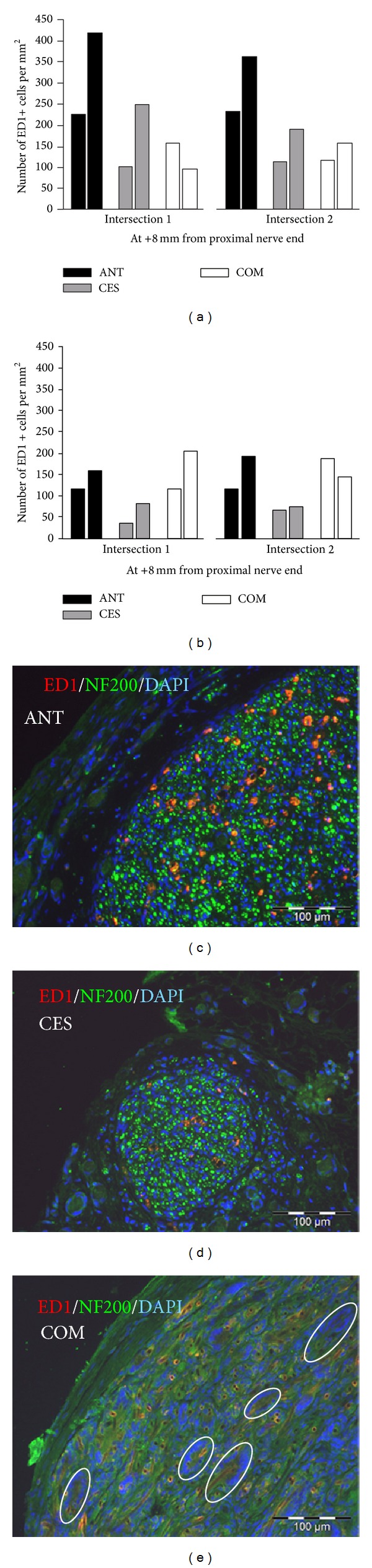
Evaluation of immunological events by immunocytochemistry. (a)-(b) Quantification of ED1-immunopositive cells, in the distal graft in two animals per group at two (a) and thirteen weeks (b) after surgery. Bars represent absolute numbers for single animals. (c)–(e) Representative photomicrographs of outer areas of graft cross-sections at 20x magnification after immunocytochemistry for ED1, NF200, and DAPI nuclear counterstaining. (c) ANT group sample containing equally distributed axons. (d) CES group sample displaying an area with equally distributed axons separated from connective tissue. (e) COM group sample demonstrating massive infiltration of the outer PCL shell with multinucleated foreign body giant cells (FBGCs, white circles), while no axons were detectable.

Anti-ED1 immunohistochemistry at 2 weeks (Figure 3(a)) after surgery revealed most ED1+ macrophages in ANTs, followed by animals with CES grafts. The smallest amount of ED1+ cells was seen in animals with COM grafts.

Thirteen weeks after surgery, the COM grafted nerves showed an expanding number of ED1+ cells, whereas for the other grafts the amount of ED1+ phagocytotic cells declined (Figure 3(b)). Anti-neurofilament 200 immunostaining showed a comparable distribution of axonal cross-sections within the CES grafts and ANTs at 13 weeks after surgery. In contrast COM grafted nerves only showed occasional nerve fibers at this point of time. Representative photomicrographs of the ANT (Figure 3(c)), CES (Figure 3(d)), and COM graft (Figure 3(e)) with ED1/NF200 immunostaining illustrate the amount and distribution of the ED1+ cells.

**Figure 4:**
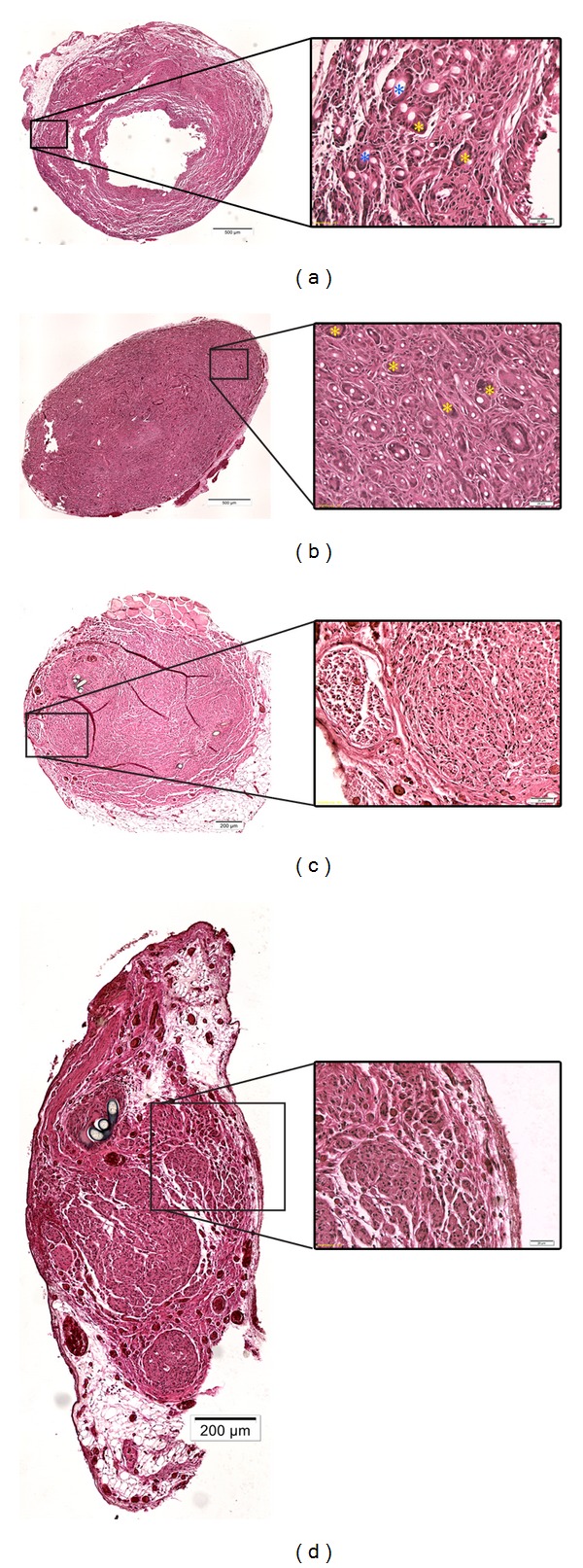
Evaluation of immunological events by HE staining. Representative photomicrographs of graft cross-sections consecutive to the sections shown in Figure 3 stained with hematoxylin and eosin. (a) COM graft, 2 weeks after implantation (dislocated inner chitosan core is not shown), (b) COM graft, 13 weeks after implantation, (c) ANT graft, 13 weeks after implantation, (d) CES graft, 13 weeks after implantation. Overview images were taken at 10x magnification (left column, scale bar (a)-(b): 500 *μ*m, (c)-(d) 200 *μ*m). Details taken at 20x magnification allow identification of FBGCs (right column, scale bars 20 *μ*m). Yellow asterisks mark FBGCs without ingested material ((a) + (b) right column), whereas blue asterisks mark FBGCs with ingested material ((a), right column).

At two weeks after surgery vast multinucleated FBGC formation invading the entire outer PCL shell and the peripheral layer of the chitosan core was found in both animals with reconstruction by COM graft (Figure 4(a); yellow asterisks). Ingested biomaterial debris was found in FBGCs and individual macrophages in these grafts (Figure 4(a); blue asterisks). The inner chitosan core was not invaded by FBGCs.

At 13 weeks after operation, the COM grafts showed extensive FBGC formation occupying the entire graft with further increased amount and size of FBGCs and individual macrophages (Figure 4(b); yellow asterisks). Only small residues of the PCL or chitosan material were detected in the extracellular space but most of the phagocytotic cells were filled with biomaterial debris. From sequential slices it was concluded that at least some of the FBGCs were ED1+ (Figure 3(e)). In contrast, in ANTs (Figure 4(c)) and CES grafts (Figure 4(d)), foreign body giant cell (FBGC) formation could only be found close to suture material without having a relation to the implanted biomaterial at two and thirteen weeks, respectively. Residues of phagocytosed biomaterial debris were not seen in these grafts.

### 3.3. Imaging of Explanted Biomaterial

**Figure 5:**
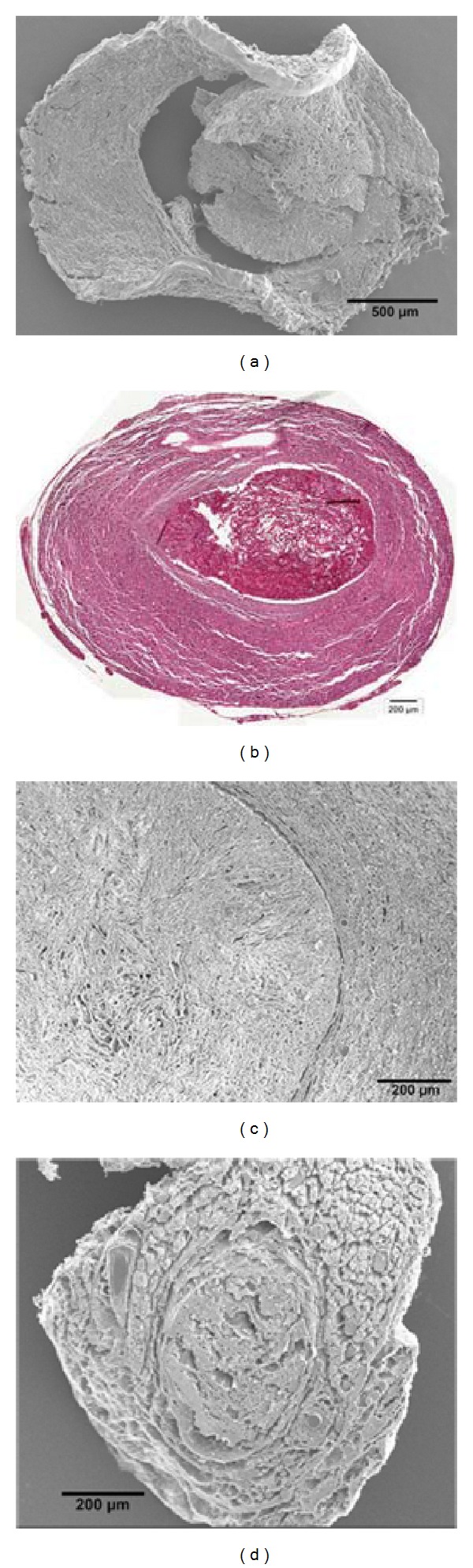
Biomaterial structure of CES and COM grafts after explanation. (a) Electron micrograph of a cross-section through a COM graft after 14 days* in vivo*, displaying only loose connection between the outer PCL shell and the inner chitosan core. (b) HE-stained cross-section demonstrating the dark colored porous chitosan core in the center ensheathed by swollen PCL shell massively infiltrated by cells. (c) Electron micrograph of a cross-section through a COM graft after 13 weeks* in vivo*, where the chitosan degradation was reduced and the pore structure still visible. (d) Electron micrograph of a cross-section through a CES graft after 13 weeks* in vivo*, no longer displaying the original core structure and firmly connected to the outer epineurium.

Analysis of the material properties two weeks after implantation revealed that in COM grafts the PCL shell was only loosely connected to the inner chitosan core. This indicates that a separation of the chitosan core from the PCL shell took place during the first two weeks after implantation as demonstrated by a circular gap between both structures (Figures 5(a) and 5(b)). Furthermore a swelling and massive cellular infiltration of the area ensheathing the chitosan core is already evident at two weeks after implantation (Figure 5(b)).

The 13 weeks after implantation SEM imaging showed that the original porous structure of the chitosan core was still detectable in COM grafts (Figure 5(c)). The PCL fiber shell and to a lower degree the chitosan core were strongly infiltrated by cells (Hoechst staining, data not shown). In contrast, CES grafts were well embedded in the epineurium (Figure 5(d)) and ensheathed by a distinct layer of cells (Hoechst staining, data not shown). Remarkably, the original porous structure of the chitosan core was no longer detectable 13 weeks after implantation directly into the epineurial sheath (Figure 5(d)). This indicates that the isolated chitosan core without PCL shell was subjected to faster degradation. This conclusion is supported by the finding that the isolated chitosan chore (CES graft) was already smaller in diameter and in degradation two weeks after implantation (data not shown). In contrast to the CES grafts, ANTs showed a sponge-like structure in SEM images with clearly distinguishable epineurium and nerve fascicles, both containing a considerable amount of Hoechst-positive cells (data not shown).

### 3.4. Pain Perception Testing (Pinch-Test)

**Figure 6:**
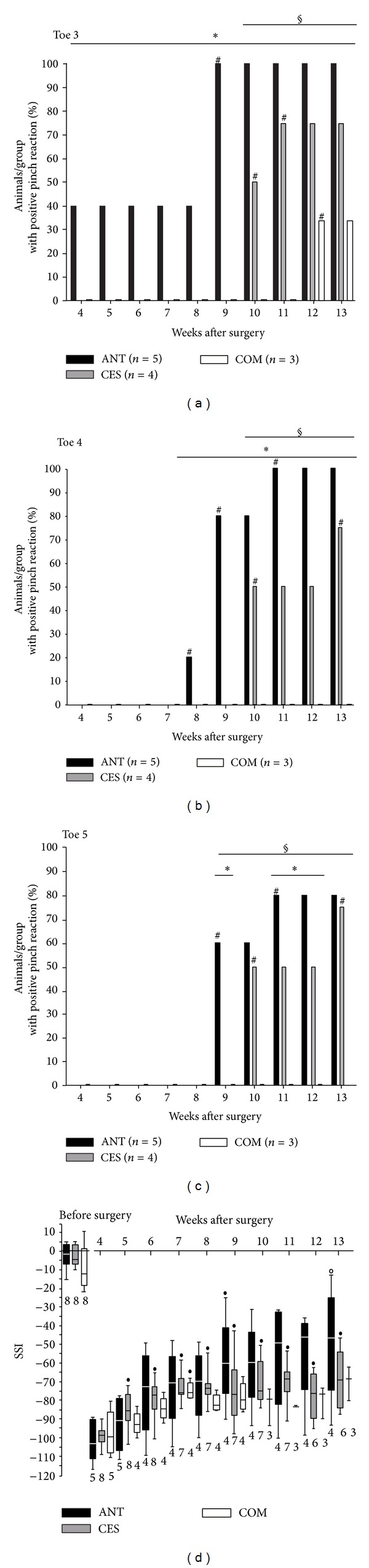
Functional evaluation of nerve regeneration. (a)–(c) Results of the pain perception test (pinch-test) performed weekly on toe III (a), toe IV (b), and toe V (c) are shown. Bars depict the percentage of animals per group with a positive pinch reaction: *intergroup difference to ANT; ^§^intergroup difference to COM graft; ^#^intragroup difference to previous week; *P* < 0.001. (d) Results of the hind limb motor function evaluation by calculation of the static sciatic index (SSI) are presented: ^•^significant intragroup improvement in comparison to 4 weeks postsurgery value; ^**o**^no longer significantly different to presurgery value; *P* < 0.05–*P* < 0.001.

To evaluate recovery of mechanoceptive pain perception, the pinch-test was performed weekly until the end of the observation period starting at 4 weeks after intervention. Not all animals could be regularly subjected to pinch-test evaluation due to signs of automutilation or death. Only results from animals that could be tested regularly during the complete observation period are shown here (ANT, *n* = 5; CES, *n* = 4; COM, *n* = 3). The percentage of animals with positive pinch reaction per group is shown in Figure 6 for the 3rd (Figure 6(a)), 4th (Figure 6(b)), and 5th (Figure 6(c)) toe. Statistical inter- and intragroup differences were analyzed with the chi-square test. The distal phalanx of the 2nd toe served as a positive control and revealed functional pain sensitivity in all of the animals for the entire trial period.

ANTs allowed the fastest recovery of mechanosensitivity with the first positive pinch reaction on toe 3 at four weeks (40%, *n* = 2 of the animals), on toe 4 at 8 weeks (20%, *n* = 1 of the animals), and on toe 5 at 9 weeks (60%, *n* = 3 of the animals) after surgical intervention. The CES graft also allowed recovery of pain perception in all toes with 50% (*n* = 2) of the animals being tested positively for all toes at 10 weeks after intervention for the first time. Animals with implanted COM graft only showed positive pinch reaction on toe 3 beginning at 12 weeks after implantation (33%, *n* = 1 of the animals).

At the end of the observation period all groups showed significant improvement (*P* < 0.001) of pain perception in toes with positive pinch reaction compared to the first postoperative values. At 13 weeks after transplantation animals with ANTs showed significantly more positive pinch reactions than animals with CES graft for toes 3 and 4 (*P* < 0.001). Pinching of toe 5 did not reveal significant differences between these groups. At the final measurements animals with implanted COM graft showed significantly fewer positive pinch reactions than the animals with other grafts (*P* < 0.001) for toes 3 to 5.

### 3.5. Evaluation of Motor Function: Static Sciatic Index

The SSI was calculated weekly by digital image analysis of the animals' plantar surface.  Figure 6(d) illustrates the results of our consecutive measurements. The animal number varies in course of our measurements due to hamstrung toe contractures, signs of automutilation, and death of two animals and is given in the graph for each week, respectively. Intragroup differences were analyzed by the Mann-Whitney test. In case of healthy sciatic nerve the preoperative static sciatic index did not differ significantly between the groups and was in accordance with values for normal motor function (SSI = 0 ± 11) given in the literature [41].

At four weeks after nerve reconstruction, animals from all groups showed significantly decreased motor function (*P* < 0.05–0.001) without intergroup difference when compared to healthy extremity motor function. These values correlate well to delineated values in lesioned hind paws (SSI = −100) [41].

A significant improvement (*P* < 0.05) of the increasing motor function in ANTs was only detected at 9 weeks after surgery compared to the first preoperative measurements (−58.87 ± 13.40; *n* = 4). At the end of the observation period SSI measurements of animals that received an ANT (−49.86 ± 16.94; *n* = 4) were no longer significantly different from preoperative values. However, due to extensive intragroup variation the improved values were also not significantly different from the first postoperative values.

Beginning 5 weeks after surgery (−85.25 ±  3.63; *n* = 8; *P* < 0.05) till the end of the observation period, animals with CES grafts showed significant recovery of motor function (varying *P* < 0.05/week) compared to first measurements after reconstruction. However, motor function remained significantly worse than preoperative values in the CES group (−69.08 ± 5.94; *n* = 6; *P* < 0.01).

Animals of the COM group showed a singular significant increase in SSI measurements at 7 weeks after transplantation (−74.69 ± 2.50, *n* = 4; *P* < 0.05) compared to first postoperative measurements. In the following weeks data was too poor to demonstrate significant differences in comparison to preoperative and first postoperative values. Overall, information on significant intergroup differences could not be obtained, although our measurements suggest best motor recovery with ANTs, followed by CES and finally COM grafts with all of them having ascending SSI values indicating motor function recovery.

### 3.6. Muscle Weight Ratio

**Figure 7:**
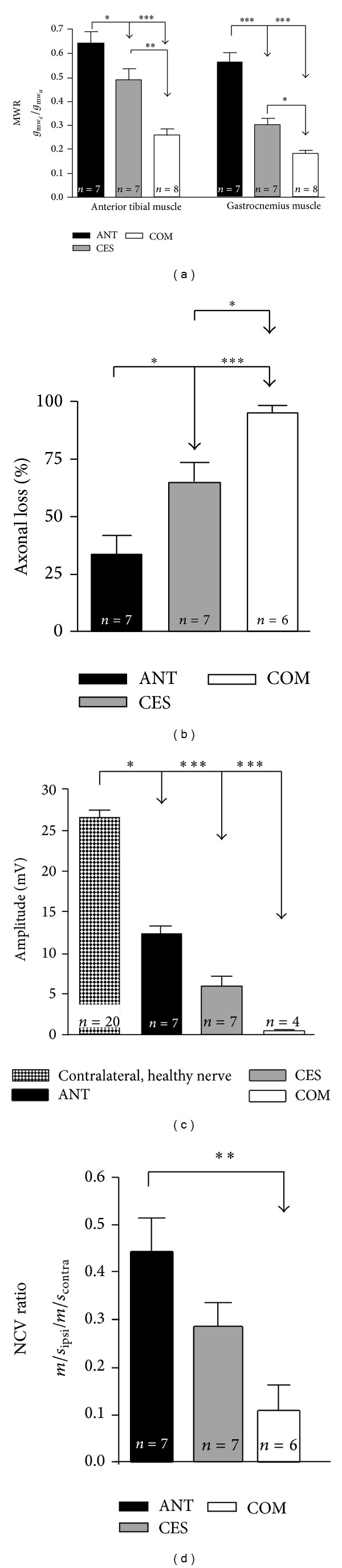
Evaluation of motor function recovery. A summary of parameters related to motor recovery evaluated 13 weeks after surgery is depicted. (a) Ratio of the operated hind limb muscle weight to the nonoperated hind limb muscle weight, (b) functional axonal loss from electrophysiological analysis (%), (c) amplitude of the maximum compound muscle action potential (M CMAP) (mV), and (d) ratio of nerve conduction velocity (NCV). Due to high current intensities only four animals of the COM group were subjected to the detailed analysis of electrophysiological properties (M CMAP amplitude). Bars represent mean ± SEM of the given data. Intergroup differences are indicated by *P < 0.05, **P < 0.01, and ***P < 0.001.

The muscle weight ratio was calculated at 13 weeks after surgery. The relative muscle weight of the operated to nonoperated lower extremity was significantly higher in animals that were treated by autografting (gastrocnemius muscle 0.56 ± 0.04, anterior tibial muscle 0.64 ± 0.05; *n* = 7) than in animals with artificial nerve transplants (CES: gastrocnemius muscle 0.30 ± 0.03; *P* < 0.001, anterior tibial muscle 0.49 ± 0.04; *P* < 0.05; *n* = 7/COM: gastrocnemius muscle 0.18 ± 0.01; *P* < 0.001, anterior tibial muscle 0.26 ± 0.03; *P* < 0.001; *n* = 8) for both, the anterior tibial and the gastrocnemius muscle (Figure 7(a)). Additionally the CES group showed significantly less muscle atrophy in both muscles compared to the COM group (*P* < 0.05–0.01).

### 3.7. Electrodiagnostic Measurements

In Figures 7(b)–7(d) results of electrodiagnostic recordings are depicted. These measurements were carried out at the end of the observation period to assess the progress of nerve regeneration. The percentage loss of functional axons (Figure 7(b)) was significantly less in ANT animals (33.76 ± 7.71%; *n* = 7) compared to CES (64.39 ± 8.71%; *n* = 7) and COM (94.99 ± 3.12%; *n* = 6) grafted animals. Additionally, the percentage of functional axons calculated was significantly higher in the CES than in the COM treated group. Analysis of maximum CMAP amplitudes (Figure 7(c)) revealed results with significantly lower amplitudes for all the experimental groups (ANT: 12.49 ± 0.97 mV; *n* = 7/CES: 6.03 ± 1.09 mV; *n* = 7/COM: 0.51 ± 0.20 mV; *n* = 4) when compared to normal nerve. There were no statistically significant intergroup differences detectable, although one could conclude from the order of magnitude of the mean values that the ANT has the highest CMAP amplitude followed by the CES and then the COM group. A detailed electrophysiological investigation was not possible in two animals of the COM group because the current intensity was too high to guarantee an isolated current conduction across the nerve. Therefore, only 4 animals were investigated. Nerve conduction velocity investigation (Figure 7(d)) showed a significantly reduced NCV ratio for the COM group (0.11 ± 0.05; *n* = 6) compared to ANT animals (0.44 ±  0.07; *n* = 7). Animals with a CES graft (0.28 ± 0.05; *n* = 7) did not show any significant difference compared to the other experimental groups.

### 3.8. Histomorphometry

**Figure 8:**
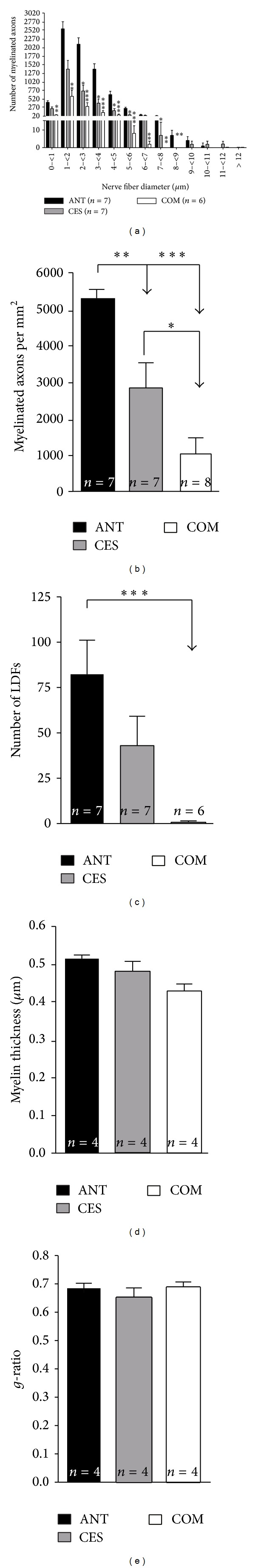
Histomorphometric analysis. The analysis of nerve fibers at +10.5 mm distal to proximal nerve end at 13 weeks after surgery is shown. (a) Myelinated nerve fiber distribution according to number of fibers per class of fiber diameters. Bars represent mean ± SEM. Significant differences to the ANT group are marked as *P < 0.05, **P < 0.01, and ***P < 0.001. (b)–(e) Results of nerve morphometry with nerve fiber density (b), number of large diameter fibers (c), myelin thickness (d), and *g*-ratio (e). Bars represent mean ± SEM of the given data. Intergroup differences are indicated by *P < 0.05, **P < 0.01, and ***P < 0.001.

Nerve grafts were resected with their adjacent nerve ends and the distal coaptation area was analyzed by histomorphometric techniques.  Figure 8 illustrates the results of the histological specimens examination. The distribution of the nerve fiber diameter (Figure 8(a)) revealed a comparable diameter distribution between the three groups especially for small diameter nerve fibers. For each axon diameter measured distal to COM grafts, we found a significantly reduced amount of axons compared to the ANTs (varying *P* < 0.05). Distal to CES grafts, we found a significant reduction in comparison to ANTs only for the amount of axons with diameters from 2 *μ*m to <6 *μ*m and from 7 *μ*m to <9 *μ*m (varying *P* < 0.05). For larger axon diameters no significant differences were detected between the CES and ANT specimens. Furthermore, the largest axon diameters were measured in CES grafts. The nerve fiber density (Figure 8(b)) of the reconstructed nerves was significantly higher in the ANT group (5298 ±  257, *n* = 7) than in nerves regenerated through CES (2907 ± 614, *n* = 7; *P* < 0.01) or COM grafts (1068 ± 400, *n* = 8; *P* < 0.001). For the artificial grafts, the CES showed a significantly higher axon density in comparison to the COM graft (*P* < 0.05). In two specimens of the COM group no myelinated nerve fibers were detected. In the specific analysis of large diameter fibers (LDFs ≥ 6 *μ*m) [42], no significant difference between the ANT (82 ± 18, *n* = 7) and the CES graft (43 ± 16, *n* = 7) was found (Figure 8(c)). Distal to the COM grafts (3 ± 3, *n* = 6) a significantly smaller number of large diameter fibers compared to ANTs were detected (*P* < 0.01; Mann-Whitney test). Although there is no significant intergroup difference between the CES and ANT or CES and COM grafts, respectively, the data indicate for CES specimens to have less regenerated LDFs than ANT specimens but more than the COM samples. Myelin sheath thickness (Figure 8(d)) is most distinct distal to ANTs (0.51 ± 0.01 *μ*m, *n* = 4). However, for the artificial grafts axonal maturation is comparable to ANT with a myelin thickness of 0.49 ± 0.03 *μ*m (*n* = 4) in CES and 0.43 ±  0.01 *μ*m (*n* = 4) in COM grafts. The *g*-ratio, as a second indicator for myelination, was best in CES specimens (0.66 ± 0.03, *n* = 4), but ANT (0.69 ± 0.01, *n* = 4) and COM samples (0.70 ± 0.01, *n* = 4) demonstrated a comparable progress of myelination without significant differences among the groups (Figure 8(e)). The maturation of myelination was evaluated in the four animals with the highest axon count of their corresponding group.

## 4. Discussion

In the present study, we developed a composite graft with an inner chitosan core plus an outer electrospun porous polycaprolactone nanofiber shell (COM). In order to assess the histocompatibility of the grafts, the presence of ED1+ macrophages and foreign body giant cells were evaluated after graft transplantation into rat sciatic nerve gaps. We found an increasing order of the amount of ED1+ macrophages from the COM over the CES to the ANT group at two weeks after surgery. With ongoing nerve regeneration and material disintegration, the number of ED1+ cells, as a sign of inflammatory reaction to the biomaterial [43], was declining in CES grafts and ANTs till 13 weeks after surgery. In contrast to this in COM grafts ED1+ cells further accumulated and contributed to the formation of FBGCs. Because NGCs made of PCL have been described as supportive for peripheral nerve regeneration [15, 16], we used this material for sheathing multichannel chitosan cores in our study and were surprised by this massive tissue reaction. Macrophages fuse to form FBGCs when biomaterial debris is of too large size to be cleared by phagocytosis [43]. The persistence of high concentrations of lytic enzymes, reactive oxygen intermediates, and the secretion of proinflammatory cytokines at the lesions site together with the presence of FBGCs could lead to failure of implanted NGCs [43]. Also other investigators propose that a porous multilayer polycaprolactone wall could demonstrate fast material degradation and thus induce massive foreign body reaction due to its high surface to volume ratio [44, 45]. Furthermore, severe FBGC formation and failure of nerve regeneration due to swelling, fibrosis, ischemia, and thus necrosis of the neural tissues were reported experimentally and clinically [44, 46]. In the present study, the remarkably massive tissue reaction and the poor performance of the COM grafts with regard to nerve recovery could additionally be attributed to the polycaprolactone shell properties. Although PCL has shown to be biocompatible and useful for tissue engineering [47], it is suggested that the dense network of PCL fibers could additionally hinder the nutrient supply of the outgrowing axons and their associated cells [44]. Although the mesh diameter for nonrotating spinning in our study was generally of favorable size for nerve grafts [48], the rotation during electrospinning of the PCL shell around the chitosan core could have induced functional occlusion of the wall. This conclusion is further supported by the lowest amount of hematogenous (ED1+) macrophages in the COM grafts, which is very likely due to insufficient neovascularization in comparison to CES and ANT grafts. A sufficient neovascularization is essential for the nutrient supply of regenerating nerves and their adjacent cells [49]. Differences in neovascularization can influence the outcome of peripheral nerve regeneration [11]. Epineural vessels, which enwrapped the CES graft, may have supported the vascularization of the grafts and therefore enabled enhanced axonal regeneration and functional recovery in comparison to COM grafts.

Regarding functional peripheral nerve recovery in the present study, the sensitivity tests revealed recovery of pain perception to a certain extent in all nerve reconstruction approaches. However, in COM grafts positive pinch reactions were detectable for toe 3 solely. This may be more attributable to collateral branching of the saphenous nerve instead of sufficient target organ reinnervation by sciatic nerve fibers [29, 50]. In contrast, animals of the ANT and CES groups also showed recovery of pinch sensitivity for toes 4 and 5. As the pinch-test response to the 5th toe did not differ significantly between them, it can be concluded that pain perception recovery was retarded in the CES group but not less complete than in the ANT group.

Motor function tests further showed an improvement in all groups. The evaluated SSI values demonstrated increasing recovery over time; however, the values nearly reached a plateau with an even declining index in some animals at the end of the observation period. This course of sciatic nerve function indices was also seen by other authors and can be explained by reinnervation of distal target muscles with functional return, but subsequent toe contractures due to inadequate target organ reinnervation [10, 51]. However, the return of motor function was clearly proven by our electrophysiological findings with measurable CMAPs in at least some animals of all grafting approaches. Of special interest is the fact that the ratios of nerve conduction velocity and the M CMAP amplitudes did not differ significantly between the CES and ANT groups. This is in line with the finding that the number of LDFs which indicate muscle reinnervation and mainly represent the A-fiber activity given in the CMAP and the progress of myelination were not significantly different among these groups [42, 52, 53]. With consideration of the muscular atrophy and the percentage loss of functional axons, target organ reinnervation is still superior in the ANT group, although in the CES group no significant impairment of motor function recovery was detected. The evidence of significantly more myelinated axons in distal nerve cross-sections corroborates the outstanding performance of the ANT compared to the artificial grafts. In contrast to healthy nerves having a bimodal distribution of axon diameters (peaks at 3.5 *μ*m and 8.5 *μ*m) [42], in the present study the amount of axons in all groups showed a single peak at 1 − <2 *μ*m with a comparable diameter distribution and sufficient myelination (*g*-ratio approximately 0.6, [37]). It can be concluded that the 3D chitosan core provides a tailored endoneural microenvironment that has no selecting influence on the distribution of the axonal diameters and allows oriented outgrowth of axons and their related SCs. In line with this conclusion, it has been demonstrated that longitudinally oriented interconnected collagen tubes manufactured by directional solidification provide a supportive scaffold for axonal regeneration [8]. The results of the presented* in vivo* experiments show that bare chitosan cores were of satisfactory chemical and mechanical stability to temporarily serve as a nerve guidance scaffold implanted into an epineural sheath (CES). Therefore, no crosslinking or neutralization of the used chitosan was necessary. Thus, the addition of chemicals with potential negative impact to the graft material can be avoided.

With regard to the massive foreign body reaction to the COM grafts as well as the poor axonal regeneration through them, it is obvious that material characteristics and degradation rate disqualify the PCL shell for fabrication of NGCs. In consideration of the chitosan core used in the present study, no signs of a foreign body response to the chitosan material were detected in our histological evaluation underlining the fact that 3D multichannel porous chitosan cores should be considered as candidate scaffolds for composite nerve grafts [12].

## 5. Conclusion

In conclusion, our results demonstrate that directional solidification and lyophilisation can be used to generate porous chitosan cores for tailored nerve guidance channels, which mimic the endoneural microenvironment of peripheral nerves. However, the type of polymer used and probably also the technique of sheathing an electrospun shell around the core scaffold have to be further investigated in the future. From the presented study we have to conclude that the outer shell enabling suture to dehiscent nerve ends should not be produced by electrospinning of polycaprolactone.

## Figures and Tables

**Table 1:** Experimental design.

	Autologous nerve transplant ANT	3D-chitosan core in epineurial sheaths CES	Composite graft (chitosan core in PCL shell) COM
Histological and material assessment	*n* = 2, week 2	*n* = 2, week 2	*n* = 2, week 2
	*n* = 2, week 13	*n* = 2, week 13	*n* = 2, week 13
Functional evaluation			
Static sciatic index, weekly	*n* = 8-4	*n* = 8-6	*n* = 8-3
Pinch test, weekly	*n* = 5	*n* = 4	*n* = 3
Electrophysiology	*n* = 7	*n* = 7	*n* = 6-4
Muscle weight ratio	*n* = 7	*n* = 7	*n* = 8
Nerve morphometry			
Nerve fiber assessment	*n* = 7	*n* = 7	*n* = 8-6
Myelin thickness and *g*-ratio	*n* = 4	*n* = 4	*n* = 4

Summary of the experimental design and illustration of the animal/specimen number (*n*) subjected to each evaluation. Varying animal numbers displayed for the evaluation of the Static Sciatic Index and the electrophysiological measurements are due to hamstrung toe contractures, signs of automutilation, and death of two animals during the course of the experiment.
